# Cross-validation of biomonitoring methods for polycyclic aromatic hydrocarbon metabolites in human urine: Results from the formative phase of the Household Air Pollution Intervention Network (HAPIN) trial in India

**DOI:** 10.1016/j.jchromb.2020.122284

**Published:** 2020-10-01

**Authors:** Naveen Puttaswamy, Sudhakar Saidam, Gayathri Rajendran, Kokila Arumugam, Savannah Gupton, Erin W. Williams, Cierra L. Johnson, Parinya Panuwet, Sarah Rajkumar, Maggie L. Clark, Jennifer L. Peel, William Checkley, Thomas Clasen, Kalpana Balakrishnan, Dana Boyd Barr

**Affiliations:** a Department of Environmental Health Engineering, Faculty of Public Health, Sri Ramachandra Institute of Higher Education and Research, Chennai, TN, India; b Gangarosa Department of Environmental Health, Rollins School of Public Health, Emory University, Atlanta, GA, USA; c Department of Environmental and Radiological Health Sciences, Colorado State University, Fort Collins, CO, USA; d Division of Pulmonary and Critical Care, School of Medicine, Johns Hopkins University, Baltimore, MD, USA; e Center for Global Non-Communicable Diseases, Johns Hopkins University, Baltimore, MD, USA

**Keywords:** Household air pollution, Polycyclic aromatic hydrocarbons, Cross-validation, 2-naphthol, 1-hydroxypyrene, HPLC-fluorescence

## Abstract

The Household Air Pollution Intervention Network (HAPIN) trial is evaluating health benefits of a liquefied petroleum gas (LPG) stove intervention in biomass cook-fuel using homes (n = 3200) in four low-and middle-income countries (LMICs) that include Peru, Guatemala, Rwanda and India. Longitudinal urine samples (n = 6000) collected from enrolled pregnant women, infants and older women will be analyzed for biomarkers associated with exposure and health outcomes. We report results from cross-validation of a lower cost high-performance liquid chromatography with fluorescence detection (HPLC-FLD) method with a higher resolution liquid chromatography-tandem mass spectrometry (LC-MS/MS) method for the measurement of 1-hydroxypyrene (1PYR) and 2-naphthol (2NAP). Twenty-five split urine samples were analyzed by HPLC-FLD method at the India trial site in Chennai, India and by LC-MSMS method at the trial wide Biomarker Coordinating Center, Emory University, USA. The limits of detection (LOD) for the HPLC-FLD method were 0.02 ng/mL and 0.07 ng/mL for 2NAP and 1PYR, respectively. Bland-Altman analysis estimated a bias of 2.98 ng/ml for 2NAP (95% CI: −5.22, −0.75) and 0.09 ng/mL for 1PYR (95% CI: −0.02, 0.21) with HPLC-FLD levels being lower than LC-MSMS levels at higher concentrations. Analyses of additional urine samples (n = 119) collected during the formative phase of the HAPIN trial in India, showed 2NAP and 1PYR levels to be consistently above the limit of quantification (LOQ) and demonstrated the applicability of the method. The HPLC-FLD method can serve as a cost-effective and reliable analytical method to measure 2NAP and 1PYR in human urine in LMICs, within and beyond the HAPIN trial.

## Highlights

• Cross-validation of analytical techniques for 1-hydroxypyrene and 2-naphthol.
• Good agreement between low cost HPLC fluorescence (HPLC-FLD) and LC-MSMS methods.
• HPLC-FLD method was highly sensitive to 2NAP compared to LC-MSMS.
• HPLC-FLD showed large bias for 2NAP with LC-MSMS at levels greater than 20 ng/mL.
• HPLC-FLD is a cost-effective and reliable analytical method for low-and middle-income countries.

## 1 Introduction

Household air pollution (HAP) is a leading risk factor for the national disease burden in India [1]. Since HAP sources, chiefly solid fuel combustion, are known to contribute anywhere between 22% and 55% of ambient air pollution levels in India [2], [3], any amount of reduction in HAP levels would not only improve ambient air quality but significantly bring down the national disease burden [4]. Cleaner cooking fuels such as liquefied petroleum gas (LPG) are believed to improve the health of household members who otherwise rely on solid biomass in many low- and middle-income countries (LMICs). To date, there have been no large-scale field trials that have tested the health benefits of an LPG intervention in households relying on solid biomass fuels.

The Household Air Pollution Intervention Network (HAPIN) trial is a randomized controlled LPG intervention trial underway in four international research collaborating (IRC) centers in India, Peru, Guatemala and Rwanda. Using a common protocol across each IRC, the trial aims to assess the health effects among pregnant women (n = 2400), children (n = 2400) and non-pregnant adult women (n = 800). More details about the HAPIN trial are described elsewhere [5]. One of the objectives of the trial is to determine the efficacy of the intervention using a battery of biomarkers that provide information about exposures, early biological effects and susceptibility among the study population. A Biomarker Center established within the Laboratory for Exposure Analysis and Development in Environmental Research (LEADER) at Rollins School of Public Health, Emory University, Atlanta, GA, USA, oversees biomarker activities across the four IRCs and provides technical guidance and support in development and validation of analytical methods in the India IRC. For further details about the rationale and design of the biomarker component of the HAPIN trial see [6].

Polycyclic aromatic hydrocarbons (PAHs) are ubiquitous pollutants generated during incomplete combustion from a variety of indoor sources. Emissions from biomass combustion are reported to generate about 5 to 10 times higher particulate phase PAHs compared to fossil fuel combustion [7]. Indoor concentrations of total particulate PAHs in a typical Indian kitchen using biomass would range between 4 and 27 µg/m^3^ which is 4–7 times as high as levels during non-cooking periods or in LPG-using households [8]. In addition, other indoor sources such as tobacco smoke, incense burning, mosquito coil use, kerosene lamps and moth repellants typical of Indian households contribute significantly to indoor PAH levels [9]. Thus, Indian women cooking with biomass fuel experience dual exposure to particulate matter and carcinogenic PAHs.

PAHs chemisorbed onto fine and ultrafine particles are known to be mutagenic, genotoxic, teratogenic and carcinogenic [10], [11]. Several molecular epidemiologic studies have quantified the effect of prenatal PAH exposure on multiple child health outcomes including low birth weight, congenital heart disease, respiratory symptoms, childhood obesity, child IQ, behavior and intelligence, and cancer risk in adulthood [12], [13], [14], [15], [16], [17], [18], [19]. While air PAH concentrations inform about environmental exposures, they do not confirm internal exposures and require extensive resources for air sampling, analysis and quality assurance and quality control (QA/QC). Urinary mono-hydroxylated PAH metabolites (OH-PAHs) provide a good integrative measure of internal exposure to PAHs and have been shown to associate with adverse birth outcomes [20], [21]. Because of biological half-lives in the range of hours to days, high specificity to parent PAHs and ease of measurement in urine, OH-PAHs are regarded as reliable biomarkers of PAH exposure in large scale population studies examining health effects of air pollution. Among the several OH-PAH metabolites, 2-naphthol (2NAP) and 1-hydroxypyrene (1PYR) are the two predominant urinary metabolites commonly detected in humans experiencing varying ranges of PAH sources and exposures [22], [23], [24], [25], [26].

A requisite in large scale human biomonitoring studies adopting OH-PAH measurements is availability of an analytical technique that is sensitive, accurate, reproducible and cost-effective. Tandem mass-spectrometry (MS/MS) or high resolution mass spectrometry (HRMS) coupled with either gas chromatography (GC) or liquid chromatography (LC) offers a powerful analytical technique with sub-picogram per mL detection capability for urinary OH-PAH measurements in the general population [22], [27]. Another widely used method is high performance liquid chromatography (HPLC) with fluorescence detection (FLD) with detection in the sub-nanogram per mL range [28], [29], [30]. While LC-MS/MS and GC-HRMS are clearly superior in detectability and specificity as compared to HPLC-FLD, availability of the instruments in laboratories with limited resources and the cost of processing thousands of samples make it difficult for LMICs to adopt these analytical techniques. Further, high PAH exposure in households using solid biomass in LMIC settings especially in India may allow the use of HPLC-FLD as a cost-effective, easy-to-adopt, reliable and adequately sensitive analytical method.

As part of the HAPIN trial, the India IRC is collecting nearly 6000 urine samples from pregnant women, adult women and children to measure urinary 2NAP and 1PYR levels using HPLC-FLD. In order to provide reliable and accurate results for the HAPIN trial, the method developed at the India IRC laboratory had to be validated. Therefore, the objective of this study was to cross-validate the HPLC-FLD method developed at Sri Ramachandra Institute of Higher Education and Research (SRU), India with the LC-MS/MS method at the HAPIN Biomarker Center at Emory University, and to apply the validated method for the analysis of 2NAP and 1PYR in urine samples collected during the formative phase of the HAPIN trial.

## 2 Materials and methods

### 2.1 Analytical standards and reagents

OH-PAH standards including 1-hydroxypyrene [1PYR (MW: 218.26 g/mol, purity 98%)] and 2-naphthol [2NAP, (MW: 144.17 g/mol, purity 99%)] were purchased from Sigma-Aldrich Inc., (St Louis, MO). HPLC-grade reagents including acetonitrile, methanol, pentane, isooctane and ethyl acetate were obtained from Fisher Scientific (Pittsburgh, PA). Sodium acetate (reagent grade, >99%), potassium dihydrogen phosphate (ACS reagent, ≥99%) and the enzyme β-glucuronidase (from *Helix pomatia*, type HP-2) was purchased from Sigma-Aldrich. Type I ultrapure water was obtained from Milli-Q water purification system (Millipore, Bedford, MA). All stock solutions were prepared by dissolving 1 mg of the native metabolites in 100 mL of methanol to obtain a 10 µg/mL concentration. Two working solutions of 2NAP and 1PYR were prepared by diluting the stock solution in methanol to yield a final concentration of either 0.5 µg/mL or 0.05 µg/mL. Calibration solutions (6 levels) were prepared in blank pooled urine matrix by spiking appropriate volumes of the working solutions to yield the following concentration ranges: 2NAP, 1–25 ng/mL and 1PYR, 0.1–6 ng/mL. All stock and working standards were prepared in amber vials and stored at −20 °C until further use.

### 2.2 Sample preparation

**Fig. 1:**
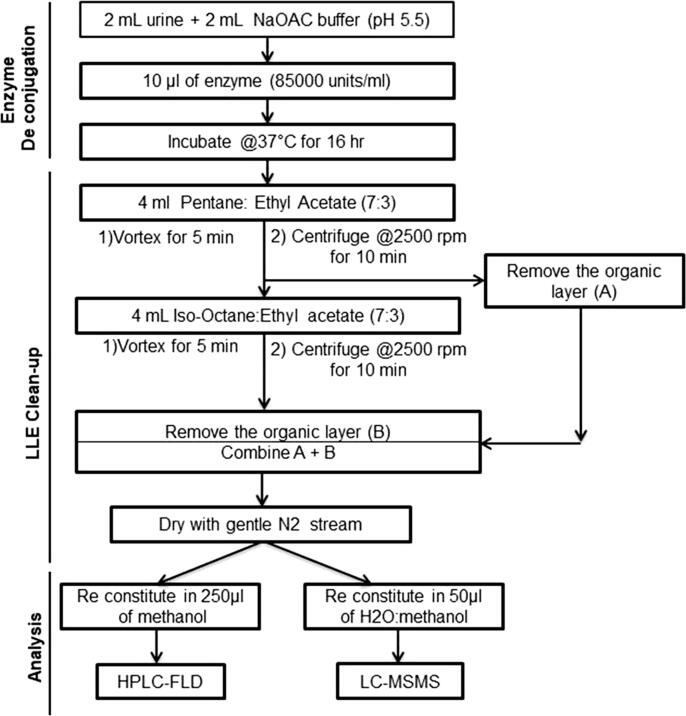
Flowchart of sample preparation and analytical method followed by SRU (HPLC-FLD) and Emory (LC-MSMS) laboratories.

Each laboratory followed a similar sample preparation protocol that broadly involved an enzymatic deconjugation step to liberate glucuronide- and sulfate-bound conjugates, liquid-liquid extraction step and analysis (Fig. 1). Prior to extraction in the LC-MS/MS method, 2 mL urine samples were spiked with a mixture of isotopically labeled analogues of the target analytes. For both methods, 2 mL of 0.2 M sodium acetate buffer (pH 5.5) and *β*-glucuronidase enzyme (derived from *Helix pomatia*; 2 mL of 2000 units/mL in LC-MSMS method and 10 µL of 85,000 units/mL in HPLC-FLD method) were added to the 2 mL aliquot of urine, and the tube was vortexed for 5 min at 1600 rpm. The mixture was incubated overnight at 37 °C for 16 h on an orbital shaker incubator at 200 rpm. To the resulting hydrolysate, 4 mL of pentane:ethyl acetate (70:30) were added and vortex mixed at 1600 rpm for 5 min followed by centrifugation at 2500 rpm for 5 min. The top organic layer was aspirated into a clean drying tube and 4 mL of *iso*-octane:ethyl acetate (70:30) was added to the remaining hydrolysate and vortex mixed for 5 min followed by centrifugation at 2500 rpm for 10 min. The top organic layer was separated and combined with the first layer in the same drying tube. The resulting organic solution was evaporated to dryness with N2 gas in a Turbovap evaporator set to 45 °C at 10 psi for approximately 30 to 45 min. The dried residues were reconstituted in 250 µL of methanol (HPLC-FLD method) or 50 µL of 1:1 MQ-water: methanol (LC-MSMS method) (Fig. 1). Reconstituted tubes were briefly vortexed for 2 min and centrifuged at 2500 rpm for 5 min. Samples were transferred to polypropylene vial inserts for analysis.

### 2.3 HPLC-FLD method

The HPLC system used was a Shimadzu LC-2010A (Kyoto, Japan) model with a 100 µL loopSIL-10ADvp auto sampler, two LC-10ADvp pumps, a CTO-10A(C) column oven, a RF-10AXL fluorescence detector and an SCL-10A system controller equipped with LC solutions software. The column used for separation of 2NAP and 1PYR was a Gemini C6-Phenyl RP column (100 × 4.6 mm I.D., 3 µm pore size, Phenomenex, Torrance, CA) with a Security Guard™ standard (4 × 2 mm, Phenomenex, Torrance, CA). The following HPLC conditions were used: eluent A, 10 mM phosphate buffer (pH 7.0) and eluent B, acetonitrile. Simultaneous determination of 2NAP and 1PYR was achieved by setting a time-gradient program as follows: 0–5 min (eluent B composition, 30%), 5–10 min (30–40% B), 10–15 min (40–60% B), 15–22 min (60% B), 22–25 min (60–80% B), 25–28 min (80% B), 28–30 min (80–30% B), 30–35 min (30% B). The excitation (Ex) and emission (Em) wavelengths were set as: 0–17 min (277/355), 17–25 min (240/387) and 25–30 min (277/355). The optimal *Ex* and *Em* wavelengths for 2NAP and 1PYR were adapted from Chetiyanukornkul et al 2006 [29]. The column temperature was set at 40 °C with a flow rate of 0.5 mL/min. The injection volume was 20 µL and the total run time was 35 min.

### 2.4 LC-MS/MS method for cross-validation

OH-PAH metabolites were measured in urine according to methods detailed elsewhere [31]. Briefly, 2NAP and 1PYR were measured in human urine by LC-MS/MS coupled with negative mode electrospray ionization (ESI) with isotope dilution quantification. During mass spectrometric analysis, the target analytes were monitored using the multiple reaction monitoring mode (MRM). One quantitation ion was monitored each for the native analyte and the labeled analog. Concentrations of the target analytes were determined from the relative response (per volume of sample injected) of native to labeled standards in the samples, by comparison to the matrix matched standard calibration curve. In each analytical run, samples are analyzed concurrently with a 10-point calibration curve, one blank sample, and two quality control samples (QCL (low level) and QCH (high level)); all prepared using 10-fold diluted pooled urine to reduce endogenous OH-PAH concentrations. The quantification range for 2NAP was 0.125–100 ng/mL. The QCL and QCH samples were prepared at a concentration of 2.50 ng/mL and 12.5 ng/mL, respectively. The quantification range for 1PYR was 0.0125–10 ng/mL. The QCL and QCH samples were prepared at a concentration of 0.50 ng/mL and 2.50 ng/mL, respectively.

The method had good precision as required by the US Food and Drug Administration guidelines (i.e., <15% relative standard deviation). During method implementation, QA/QC procedures were strictly followed. In addition, method accuracies were validated by analyses of certified standard reference material (SRM) 3672 obtained from the U.S. National Institute of Standards and Technology (NIST). The accuracies for 2NAP and 1PYR are 99% and 89%, respectively. This method was evaluated, certified, and recertified semi-annually by successful participation in the German External Quality Assessment Scheme for Analyses in Biological Materials (G-EQUAS).

### 2.5 Method validation and quality control

Twenty five de-identified human urine samples were shipped to SRU laboratory, India, from the Emory laboratory for method cross-validation. Samples were shipped frozen on dry ice and were stored at −70 °C until further analysis. Each method was run independently using similar sample preparation protocols with sample concentrations blinded to the analysts. The SRU laboratory in India used HPLC-FLD for analysis and the LEADER laboratory at Emory University used LC-MSMS for analysis to quantify 2NAP and 1PYR in these split urine samples.

For the HPLC-FLD method, blank pooled urine matrix was prepared by combining 1 mL each from 25 split urine samples and passing it 3 times through three different Bond Elut™ C18 SPE cartridge (500 mg, Agilent, Santa Clara, CA) at 1 mL/min to remove endogenous PAH metabolites. Three replicates of pooled urine sample were run in HPLC and no peaks were detected for 2NAP and 1PYR. Eight aliquots of pooled urine were made and used for preparing matrix-matched calibration standards (6 levels) and urine blanks. Calibration solutions were prepared by spiking appropriate volumes of individual standards (i.e. 2NAP and 1PYR) into 3 mL of pooled urine prior to enzymatic hydrolysis, extraction and analysis. The range of calibration standards prepared for each metabolite was: 1–25 ng/mL for 2NAP and 0.1–6 ng/mL for 1PYR. Similar levels of calibrants were prepared in methanol and each calibrant was run three times to confirm the mean retention times (Rt) which were 14.25 ± 0.05 min and 20.30 ± 0.05 min for 2NAP and 1PYR, respectively. Urine and solvent blanks were run three times to check for analyte peaks and carry-over.

QC materials were prepared by spiking standard solution into pooled urine to yield a final concentration of 1.5 ng/mL (low), 4.5 ng/mL (medium) and 15 ng/mL (high) for 2NAP and 0.1 ng/mL (low), 0.6 ng/mL (medium) and 2 ng/mL (high) for 1PYR, respectively. Accuracy was determined by analyzing replicate QC samples (n = 10) at each level and method accuracy was assessed as relative recovery and calculated as follows: (mean observed concentration/spiked concentration) × 100 (%). Intra- and inter-day precisions were calculated from observed concentrations derived from repeat measurements of the medium QC sample during a 10-day period and expressed as the percent relative standard deviation (%RSD). Each analytical batch of unknown samples contained solvent blank, urine blank, solvent calibrators, matrix-matched calibrators and three levels of in-house QC samples. The blanks and QCs were analyzed after every 10 unknown samples and QCs were considered acceptable if the calculated values fell within ±20% of spiked concentration. The calibration curves were plotted using peak area versus the six different calibrant concentrations over a linearity range of three orders of magnitude with a mean error of 0.296 and 0.102 for 2NAP and 1PYR, respectively and a correlation coefficient greater than *R* > 0.999. The limits of detection (LODs) were estimated from the linear regression curve prepared for each analyte in the pooled urine matrix as 3 times the standard deviation (σ) of the low calibrant divided by the slope of the best fit line.

### 2.6 Sample collection, storage and analysis

Formative research studies were conducted in the first year of the HAPIN trial to (i) select field sites suitable for the trial and (ii) to field test the study protocols. Since each IRC had unique field characteristics and logistical challenges, it was important to field test the biosample collection, processing and storage procedures. All four IRCs followed a harmonized sample collection protocol developed by the Biomarker Core at Emory University. HAPIN field staff at each IRC received extensive training and evaluation in biosample collection procedures to ensure sample integrity.

In India, two rural field sites were selected where communities primarily depend on solid biomass fuel for their cooking needs and contribution from ambient pollution was minimal. The Kalvarayan hills (11.704°N, 78.735°E) in Villupuram district and Thiruvarur (10.525°N, 79.636°E) in Nagapattinam district of Tamilnadu state in southern India are approximately at 250 km and 400 km from SRU laboratory. Subjects were recruited in formative research under two phases; phase I involved adult non-pregnant women (A) and children (C) less than 1 year of age both living in the same household and phase II involved pregnant women (P) from different households. Early morning urine voids were collected from P (n = 40) and A (n = 40) and spot samples from C (n = 39) to coincide with planned personal PM2.5 exposure monitoring. To maintain the cold-chain, temperature controlled Credo™ ProMed portable cooler bags (Pelican BioThermal, Plymouth, MN) were used for sample collection and transport. Aliquots were prepared in the field laboratory and stored in a −20 °C freezer. Frozen aliquots were then shipped to the central laboratory at SRU in Chennai on dry-ice and stored at −70 °C until further analysis. Field blanks and transport blanks were used to check for potential contamination during collection, handling and transport. Urine creatinine was measured spectrophotometrically using a semi-automatic biochemistry analyzer (Chem 7, Erba Mannheim, London, UK) and specific gravity was measured using a urine refractometer PAL-10S (Atago Co. Ltd., Tokyo, Japan). A case report form was administered at the time of urine collection to capture household exposure sources, potential second hand smoke exposure, cooking practices, behavior and nutrition. Aliquoted tubes were thawed at room temperature and the levels of 2NAP and 1PYR were determined following the validated HPLC-FLD method. Samples, calibrants, blanks and QC materials were analyzed by both methods as outlined above.

### 2.7 Data analysis

Method parameters such as linearity, limit of detection, accuracy and precision were estimated using Microsoft Excel and all other statistical analyses were performed using SigmaPlot 14 software (Systat Software Inc., San Jose, CA). The agreement between the HPLC-FLD and LC-MSMS was evaluated by Bland-Altman analysis. Bias and standard error of regression (SER) were calculated to understand the magnitude of disagreement between the two methods [32]. Bland-Altman plots were prepared to identify outliers and to identify any trend in the mean difference of the two methods [32]. Urinary 2NAP and 1PYR concentrations were normalized to urinary creatinine and specific gravity levels. Measures of central tendency were evaluated by calculating median and mean.

## 3 Results

**Fig. 2:**
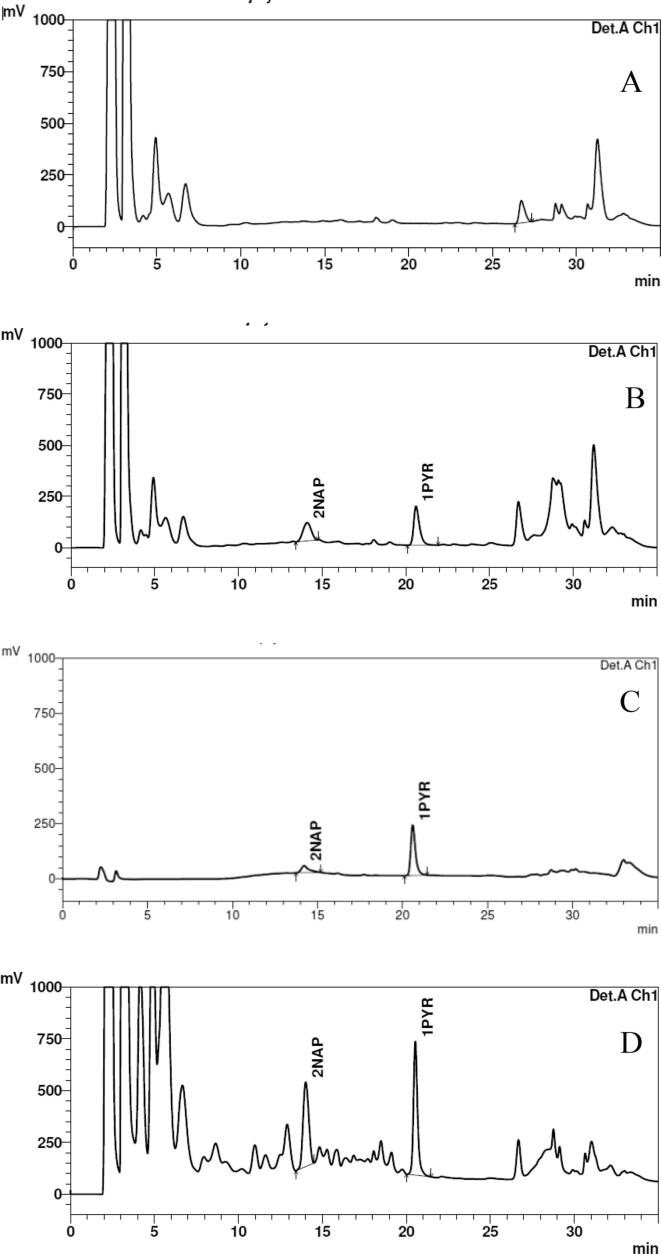
Representative chromatograms from HPLC-FLD analytical method for (a) pooled urine blank, (b) spiked urine matrix, (c) standard in acetonitrile and (d) urine sample.

In this work, urinary levels of 2NAP and 1PYR in blinded split human urine samples determined by the HPLC-FLD method at the SRU laboratory in India were compared to a highly selective and sensitive MS/MS-based analytical method developed at the LEADER laboratory in the Emory University. In addition, a relatively inexpensive liquid-liquid extraction procedure developed by LEADER laboratory was reproduced in the SRU laboratory (Fig. 1). For the HPLC-FLD method, the chromatogram of a blank, spiked urine matrix and urine sample showed good separation with no interfering peaks and a run time of 35 min (Fig. 2). The reliability of the HPLC-FLD method was demonstrated by comparing its performance to the LC-MSMS method (Table 1). The LOD for 2NAP and 1PYR in the HPLC-FLD method was ten times higher compared to LC-MSMS method and the correlation coefficient of calibration curves for both analytes was greater than 0.99 (Table 1). The intra- and inter-day precisions reported as %RSD was below 3% for both 2NAP and 1PYR. The relative accuracy tested at three different levels showed good recovery for both analytes except at low levels for 1PYR (Table 1).

**Table 1:** Method parameters of the LC-MSMS and HPLC-FLD analytic method. NA = not applicable.

Analytic method	Analyte	Quantification		Accuracy (%)			Precision (%RSD)	
		LOD (ng/ml)	R^2^	Low level	Medium Level	High Level	Intra-day	Inter-day
LCMSMS	2NAP	0.125	0.999	98.96	NA	NA	0.9	1.2
	1PYR	0.012	0.999	89.47	NA	NA	1.9	1.1

HPLC-FLD	2NAP	0.020	0.999	109.72	101.62	104.21	2.69	1.58
	1PYR	0.069	0.999	128.07	112.76	110.87	2.35	1.86

Regression analysis of all 25 samples for 2NAP gave a slope of 0.61 and an R^2^ value of 0.74 (Table 2). However, the slope and R^2^ value improved significantly towards unity when two outliers were excluded from the analysis (Table 2). For 1PYR regression analysis, neither the slope nor the R^2^ value were close to unity but with removal of outliers the R^2^ value improved from 0.06 to 0.63 (Table 2). However, the slope factor of 0.42 for 1PYR was not close to one (Table 2). We observed substantial variability in 1PYR levels close to the LOD in the HPLC-FLD method. The standard error of the regression (SER) decreased significantly for both 2NAP and 1PYR when outliers (1.5 × IQR) were excluded from regression analysis which was more evident for 1PYR compared to 2NAP (Table 2).

**Table 2:** Regression and Bland-Altman analysis parameters showing comparability of two methods.

Statistic	2NAP		1PYR	
	All data (n = 25)	Outlier removed (n = 22)	All data (n = 25)	Outlier removed (n = 23)
*Regression*				
Slope	0.61	0.749	2.03	0.42
Intercept	1.18	0.456	0.10	0.02
R^2^	0.73	0.845	0.06	0.63
p-value	0.00	<0.001	0.25	<0.001
SER	5.34	2.489	0.21	0.06

*Bland-Altman*				
Bias	−2.986	−1.580	0.093	0.097
SD	5.41	3.085	0.280	0.127
Limits of agreement	−13.583, 7.610	−7.627, 4.467	−0.456, 0.634	−0.152, 0.347

**Fig. 3:**
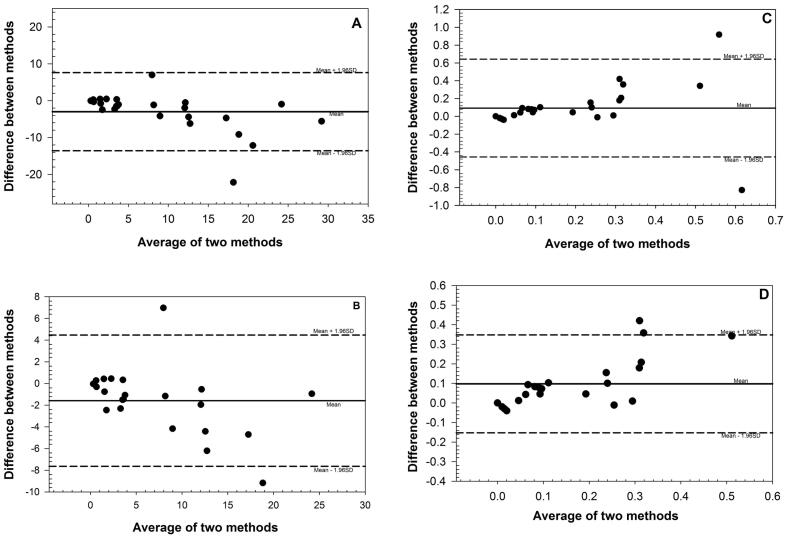
Bland-Altman plots for comparison of LC-MSMS and HPLC-FLD methods of measurement for 2NAP (A) all data points (n = 25), (B) without outliers (n = 22), and 1PYR (C) all data points (n = 25), (D) without outliers (n = 23).

**Fig. 4:**
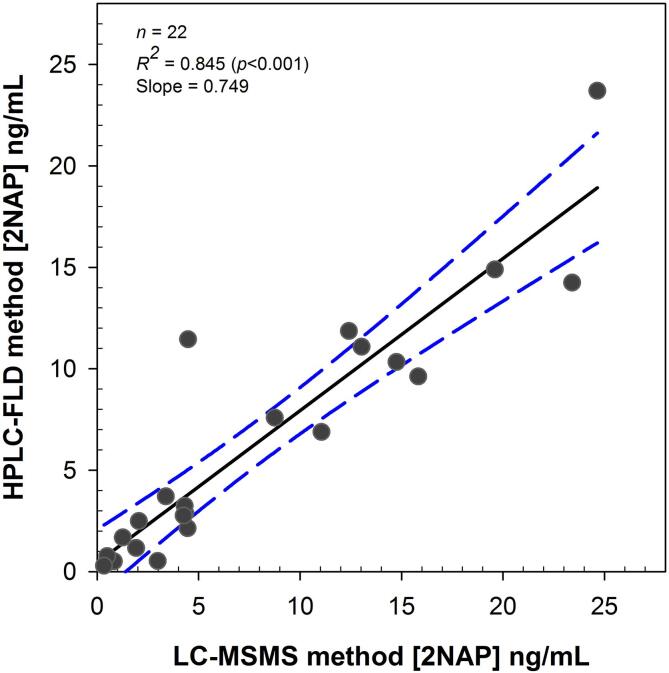
Regression analysis of 2NAP for values less than 25 ng/mL between LC-MSMS and HPLC-FLD methods.

Results from the Bland-Altman analysis for 2NAP showed that the 95% confidence intervals were fairly large and the HPLC-FLD method had a negative bias of about 2.9 ng/mL compared to the LC-MSMS method (Table 2 and Fig. 3a). Except for one value, all 24 data points were within the 95% confidence intervals (Fig. 3a). When outliers were excluded, bias decreased to about 1.5 ng/mL with much tighter 95% confidence intervals (Table 2 and Fig. 3b). We observed significant agreement between LC-MSMS and HPLC-FLD methods for 2NAP (*p* < 0.001) for values lower than 25 ng/mL (Fig. 4). For 1PYR, the limits of agreement were much tighter with a smaller positive bias (0.09 ng/mL) and SER showing a good agreement between the two methods (Table 2 and Fig. 3c). With outliers excluded, the bias remained close to 0.09 whereas the SER value decreased from 0.28 to 0.13 (Table 2 and Fig. 3d). 1PYR data was randomly distributed independent of its concentration and except for one data point, all 24 data points were within the 95% confidence intervals (Figs. 3c and 1d).

### 3.1 Method application for analysis of HAPIN formative samples

**Fig. 5:**
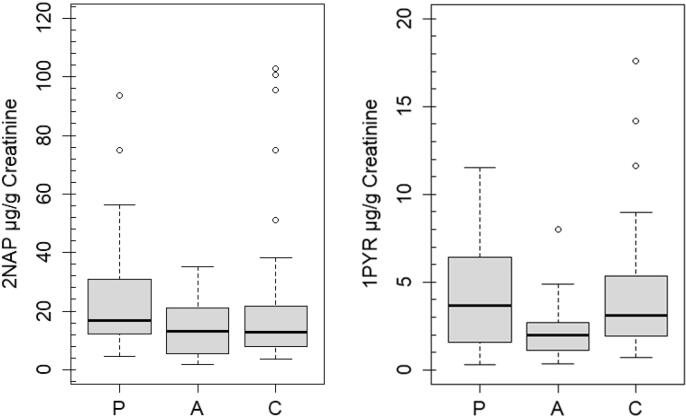
Concentrations of 2NAP and 1PYR in pregnant women (P), adult women (A) and child (C) analyzed using validated HPLC-FLD method.

Concentrations of 2NAP and 1PYR were above the LOD and LOQ in all samples analyzed across all subjects. Creatinine and specific gravity adjusted and unadjusted concentrations of 2NAP and 1PYR are given in Table 3. Unadjusted and specific gravity adjusted concentrations showed good similarity for both 2NAP and 1PYR compared to creatinine adjusted levels (Table 3). Generally, concentrations of 2NAP were 4 to 6 times higher compared to 1PYR in all samples tested (Table 3). The median concentration of 2NAP among pregnant women, adult women and children were 9.41 µg/L, 6.32 µg/L and 2.05 µg/L, respectively (Table 3). The spread of 2NAP and 1PYR levels was small among adult women followed by pregnant women and children (Table 3 and Fig. 5).

**Table 3:** Concentrations of 2NAP and 1PYR with different normalization factors for pregnant women (P), adult women (A) and child (C).

Subject	Normalization	Unit	2NAP				1PYR			
			AM	Median	GM	Min.-Max	AM	Median	GM	Min.-Max
Pregnant women (n = 40)	Unadjusted	µg/L	11.99	9.41	9.61	2.41–49.69	2.33	1.53	1.62	0.30–8.79
	Sp. Gravity^a^	µg/L	12.82	11.24	10.33	2.68–70.98	2.36	1.63	1.74	0.20–7.99
	Creatinine	µg/g	24.48	16.77	19.02	4.69–93.69	4.51	3.66	3.21	0.32–11.54

Adult women (n = 40)	Unadjusted	µg/L	7.37	6.32	5.78	0.85–31.20	1.25	1.02	0.98	0.17–4.98
	Sp. Gravity	µg/L	9.04	7.23	6.70	0.95–28.46	1.52	1.10	1.13	0.19–8.53
	Creatinine	µg/g	14.15	13.03	10.80	1.81–35.22	2.20	2.01	1.82	0.36–7.99

Child (n = 39)	Unadjusted	µg/L	3.70	2.05	2.15	0.60–29.12	0.69	0.39	0.45	0.12–6.83
	Sp. Gravity	µg/L	3.38	1.62	1.93	0.45–26.54	0.57	0.51	0.41	0.07–3.00
	Creatinine	µg/g	23.08	12.76	15.06	3.51–102.8	4.22	3.10	3.16	0.73–17.61

a Specific Gravity (SG) correction = (ng/mL) * (Median SG – 1)/(SG of sample – 1).

## 4 Discussion

The overall objective was to cross-validate the HPLC-FLD method developed at SRU laboratory in India, which is inexpensive and easy to adopt in laboratories with limited sophisticated analytical instrumentation, with a highly sensitive and superior analytic method such as the tandem mass-spectrometry. The validated HPLC-FLD method was then used to determine levels of 2NAP and 1PYR in human urine samples collected from pregnant women, adult women and children as part of the formative phase biomonitoring activities of the ongoing HAPIN trial. A total of 6000 urine samples is being collected from pregnant women (n = 800), children (n = 800) under 1 year of age and adult women (n = 200) in the ongoing HAPIN trial for the analysis of 2NAP and 1PYR. This is the first population level human biomonitoring study in India that aims to assess PAH exposure by measuring urinary levels of 2NAP and 1PYR. Therefore, an analytical method that is easy to adopt, cost effective (e.g., $30k HPLC vs. $300k LC-MSMS) and assures good accuracy and reproducibility is essential to process such large volumes of samples. In addition, 2NAP and 1PYR selected for the HAPIN trial represents inhalation and ingestion pathways of exposure to PAHs in humans. The conjugated forms of 2NAP and 1PYR are the two predominant OH-PAHs and constitute more than 95% compared to free forms in urine and are stable for more than a year when stored under −20 °C [24]. Because PAH metabolites are highly sensitive to fluorescence detection, the HPLC-FLD is a cost-effective and easy to adopt analytical technique in the absence of LC-MSMS system to reliably determine levels of PAH metabolites in large scale human biomonitoring studies such as the HAPIN trial.

### 4.1 Cross-validation performance

Each laboratory validated the method independently following standard protocols and method cross-validation was performed based on analysis of split human urine samples. Regression and Bland-Altman analysis was performed to evaluate agreement between the two analytical techniques. The HPLC-FLD method had a better sensitivity for 2NAP than LC-MSMS method. But the LOD for 1PYR was 5× higher in HPLC-FLD compared to the LC-MSMS method. The LODs obtained from the HPLC-FLD method were in good agreement with LOD values of 0.014 ng/mL for 2NAP and 0.030 ng/mL for 1PYR as reported in [28]. The sensitivity and specificity of the HPLC-FLD method is adequate to capture the 2NAP and 1PYR levels in the Indian sub-population. This is because (i) PAH emission sources are ubiquitous and India ranks 2nd in global emissions of PAHs, (ii) PAH exposure levels are among the highest in rural kitchens using biomass fuel in India and (iii) 2NAP and 1PYR are the two major OH-PAHs detected in urine of Indian adult population compared to seven Asian population [9], [33], [34], [35].

Each laboratory employed a similar sample preparation protocol via liquid-liquid extraction (LLE) developed by Emory laboratory for the simultaneous determination of OH-PAHs, malondialdehyde (MDA), and 8-Oxo-2′-deoxyguanosine (8-Oxo-dG) in urine by HPLC-MSMS [31]. Although solid phase extraction (SPE) is preferred over LLE for urine clean-up and extraction in most OH-PAH analytical procedures, we adopted LLE because of its ease of handling, efficiency of extraction and cost-efficiency to process over 5000 urine samples in the HAPIN trial. The HPLC-FLD method is set-up to process 60 samples per day per analyst (including blanks and QC samples) making it an ideal throughput process to handle large volumes of HAPIN trial samples. The LLE method resulted in an accuracy that ranged from 101% to 128% which was similar or even better compared to SPE methodology used in previous studies [22], [28], [29], [36], [37]. The inter-day and intra-day precision of pooled urine matrix spike was under 5% RSD in HPLC-FLD method which was comparable with LC-MSMS and GC-HRMS techniques [22], [37].

Agreement between the HPLC-FLD method and the LC-MSMS method was assessed using regression and Bland-Altman analysis. In general, there was good agreement in measured 2NAP and 1PYR concentrations between the two methods. The two statistical evaluations revealed interesting differences for 2NAP and 1PYR results between the two methods. Regression parameters (i.e. slope and intercept) are affected by the range of the observations, especially small sample size can influence the bias making it difficult for comparing two methods [32]. In the case of 2NAP, regression analysis of all 25 samples showed an agreement of 75% based upon the slope and a high SER of 5.34 between the two methods. When 2NAP values above 20 ng/mL was removed from analysis, the bias attributed to high 2NAP decreased by 50% and comparability improved significantly between the two methods. We speculate that the fluorescence emission intensity (the conjugate acid) drops at high 2NAP concentrations due to excited-state deprotonation by hydrogen phosphate (used as buffer) forming a conjugate base (2-naphtholate) which is not detected at the set emission wavelength resulting in low intensity [38]. This affects the accuracy and precision at high 2NAP concentrations. For concentrations above 20 ng/mL we can dilute the samples and re-analyze to get it into the range of reliable analysis. For 1PYR, regression did not support comparability again due to the small range of observations clustered at the lower end of the concentration range. On contrary, Bland-Altman analysis showed good agreement with a minor bias attributed to low concentration range of 1PYR. Regardless, by documenting the agreement and bias between the two methods, we can consider these values when combining data from the two laboratories for the HAPIN study.

### 4.2 Urinary PAH metabolites in HAPIN formative subjects

By using the validated HPLC-FLD method, 2NAP and 1PYR were quantified in urine samples collected from pregnant women (n = 40), adult women (n = 40) and children (n = 39) <1 year of age. Since urine collected from children was generally low (10 to 30 mL), mothers were instructed not to combine multiple spot samples. This is because urine volume is highly variable among different age groups and it greatly affects the concentration of urinary biomarkers within and among individuals. In order to account for variable urine volume and dilution, biomarker levels in population studies are often normalized to a constant creatinine concentration [39]. In this study, urinary 2NAP and 1PYR levels were corrected for urine dilution using specific gravity and creatinine to assess the variability among subject groups. In general, unadjusted, specific gravity and creatinine corrected 2NAP and 1PYR concentrations showed less variability for pregnant and adult women. However, creatinine correction resulted in elevated 2NAP and 1PYR levels in children by a factor of about 10 compared with un-adjusted concentrations. Creatinine levels in children are generally low compared to adults due to low fat-free mass and correction using a low creatinine number results in elevated biomarker levels for children compared to adults [39]. Such elevated levels will have strong implications on *exposure–response* models in the HAPIN trial. Therefore, to overcome such overestimations in urinary biomarker levels, creatinine-adjusted levels will be compared with a reference range derived from similar age group in this case children under 1 year of age or creatinine can be used as an independent variable in the models as suggested by [39].

The HPLC-FLD method had sufficient sensitivity to detect 2NAP and 1PYR levels in all the 119 urine samples. The minimum level detected for 2NAP in P, A and C was 2.41 ng/mL, 0.85 ng/mL and 0.60 ng/mL, respectively. These levels were about 2 orders of magnitude high compared to the method LOQ of 0.066 ng/mL for 2NAP. For 1PYR, the minimum level detected was 0.3 ng/mL, 0.17 ng/mL and 0.12 ng/mL which were 2 to 5 times higher than LOD value of 0.069 ng/mL. Pregnant women showed high 2NAP and 1PYR (un-adjusted) followed by adult women and children. This result was expected since all pregnant women reported cooking with solid biomass fuel on the previous day of urine collection. In addition, adult women who cooked the previous day had 3 times higher levels of 2NAP compared to those who did not cook. Cooking with solid biomass result in high exposures to PAHs compared to those using cleaner fuels such as the LPG. For instance, concentrations of six carcinogenic PAHs measured at the breathing zone ranged from 4.3 to 26.8 µg/m^3^ during a 2 h cooking event with solid biomass compared to 1.97–10.87 µg/m^3^ for LPG use [8]. In addition, several other indoor combustion sources such as exposure to tobacco smoke, kerosene use for lighting, incense burning and mosquito coil contribute significantly to PAHs. In many homes across India such sources of incomplete combustion contribute to high indoor PAH levels [9], [40]. Although such high exposures in rural kitchens are a common sight in India, there have been no studies to date that have assessed urinary PAH metabolites in the general population in India. In the present study, households were selected such that the contribution from ambient pollution (e.g. traffic related air pollution) was very minimal and the predominant source of particulate matter (PM2.5) and PAHs were from household combustion sources. Therefore, the urinary 2NAP and 1PYR assessed in the present study are primarily a result of exposure to indoor combustion sources.

In an effort to understand the magnitude of exposures among the Indian sub-population, urinary levels of 2NAP and 1PYR from this study were compared with values reported in studies from other countries. It was found that pregnant women and adult women in India experience some of the highest exposures to PAHs in indoor environments as evident by high levels of 2NAP and 1PYR (Table 4). To date there has been no population level human biomonitoring study in India that explicitly addressed exposures to PAHs via urinary biomarker analysis. The HAPIN trial aims to generate some of the first population level estimates of exposure to PAHs by assessing urinary levels of 2NAP and 1PYR in pregnant women, adult women and children. Therefore, adopting a validated analytical method for the measurement of PAH metabolites in the HAPIN trial ensures high quality data.

**Table 4:** Concentrations of 2NAP and 1PYR reported from different countries compared to India’s sub-population (present study).

Country	Sub-population	Size	Statistic	Unit	2NAP	1PYR	Reference
USA	Pregnant women	200	GM	µg/L	0.68	0.12	[36]
Canada	Pregnant women	57	Mean	µg/g Cr.	2.61	0.14	[25]
USA	Pregnant women	200	Median	µg/L	1.97	0.10	[41]
Puerto Rico	Pregnant women	50	Median	µg/L	5.34	0.25	
Korea	Adult women	3218	GM	µg/L	2.77	0.12	[42]
Iran	Adult women	92	GM	µg/L	2.69	0.28	[43]
USA	Adult	1625	GM	µg/L	2.64	0.05	[22]
Poland	Children	86	GM	µg/L	2.92	0.14	[44]
China	Adult	84	Mean	µg/L	6.96	0.67	[34]
Vietnam	Adult	23	Mean	µg/L	15.31	0.64	
India	Adult	38	Mean	µg/L	8.71	0.69	
Malaysia	Adult	29	Mean	µg/L	2.85	0.19	
Korea	Adult	60	Mean	µg/L	9.98	0.17	
Kuwait	Adult	38	Mean	µg/L	1.11	0.32	
China	Children	51	Median	µg/L	3.3	1.5	[45]
USA	Children	387	GM	µg/L	2.48	0.05	[22]
Poland	Children	218	GM	µg/g Cr.	8.45	0.36	[46]
India	Pregnant women	40	GM	µg/L	9.61	1.62	*Present study*
India	Adult women	40	GM	µg/L	5.78	0.98	
India	Child (<1 year)	39	GM	µg/L	2.15	0.45	

## 5 Conclusion

As part of the ongoing HAPIN trial, we undertook cross-validation of HPLC-FLD method for the measurement of 2NAP and 1PYR developed at the India IRC with LC-MS/MS technique developed at the LEADER laboratory, Emory University. Validation parameters (LOD, accuracy and precision) of the HPLC-FLD method were comparable with LC-MSMS method. Bland-Altman analysis showed large 95% confidence levels for 2NAP with HPLC-FLD data when concentrations were higher than 20 ng/mL. These samples can be diluted and re-analyzed to improve the reliability. For 1PYR, the 95% confidence levels were fairly small with good agreement between two methods. The validated method was used to measure 2NAP and 1PYR levels in urine samples collected from pregnant women, adult women and children less than 1 year during the formative phase of HAPIN trial. The HPLC-FLD method sensitivity adequately captured the minimum levels among all formative subjects. Pregnant women had high levels of 2NAP compared to adult women and children. The HPLC-FLD method can serve as a cost-effective and reliable analytical method to measure 2NAP and 1PYR in an LMIC laboratory and support biomonitoring activities of the HAPIN trial in India.

## CRediT authorship contribution statement

**Naveen Puttaswamy:** Conceptualization, Methodology, Formal analysis, Writing - original draft, Writing - review & editing. **Sudhakar Saidam:** Formal analysis, Investigation. **Gayathri Rajendran:** Investigation. **Kokila Arumugam:** Investigation. **Savannah Gupton:** Investigation. **Erin W. Williams:** Investigation. **Cierra L. Johnson:** Investigation. **Parinya Panuwet:** Methodology, Formal analysis, Writing - review & editing. **Sarah Rajkumar:** Methodology, Writing - review & editing. **Maggie Clark:** Methodology, Writing - review & editing. **Jennifer L. Peel:** Funding acquisition, Writing - review & editing. **William Checkley:** Funding acquisition, Writing - review & editing. **Thomas Clasen:** Funding acquisition, Writing - review & editing. **Kalpana Balakrishnan:** Funding acquisition, Writing - review & editing. **Dana Boyd Barr:** Conceptualization, Methodology, Formal analysis, Writing - review & editing, Funding acquisition.

## Declaration of Competing Interest

The authors declare that they have no known competing financial interests or personal relationships that could have appeared to influence the work reported in this paper.
